# Scientific research on animal biodiversity is systematically biased towards vertebrates and temperate regions

**DOI:** 10.1371/journal.pone.0189577

**Published:** 2017-12-14

**Authors:** Mark A. Titley, Jake L. Snaddon, Edgar C. Turner

**Affiliations:** 1 Insect Ecology Group, University Museum of Zoology, Cambridge, Downing Street, Cambridge, United Kingdom; 2 Biological Sciences, Institute for Life Sciences, University of Southampton, Southampton, United Kingdom; Tierarztliche Hochschule Hannover, GERMANY

## Abstract

Over the last 25 years, research on biodiversity has expanded dramatically, fuelled by increasing threats to the natural world. However, the number of published studies is heavily weighted towards certain taxa, perhaps influencing conservation awareness of and funding for less-popular groups. Few studies have systematically quantified these biases, although information on this topic is important for informing future research and conservation priorities. We investigated: i) which animal taxa are being studied; ii) if any taxonomic biases are the same in temperate and tropical regions; iii) whether the taxon studied is named in the title of papers on biodiversity, perhaps reflecting a perception of what biodiversity is; iv) the geographical distribution of biodiversity research, compared with the distribution of biodiversity and threatened species; and v) the geographical distribution of authors’ countries of origin. To do this, we used the search engine *Web of Science* to systematically sample a subset of the published literature with ‘biodiversity’ in the title. In total 526 research papers were screened—5% of all papers in *Web of Science* with biodiversity in the title. For each paper, details on taxonomic group, title phrasing, number of citations, study location, and author locations were recorded. Compared to the proportions of described species, we identified a considerable taxonomic weighting towards vertebrates and an under-representation of invertebrates (particularly arachnids and insects) in the published literature. This discrepancy is more pronounced in highly cited papers, and in tropical regions, with only 43% of biodiversity research in the tropics including invertebrates. Furthermore, while papers on vertebrate taxa typically did not specify the taxonomic group in the title, the converse was true for invertebrate papers. Biodiversity research is also biased geographically: studies are more frequently carried out in developed countries with larger economies, and for a given level of species or threatened species, tropical countries were understudied relative to temperate countries. Finally, biodiversity research is disproportionately authored by researchers from wealthier countries, with studies less likely to be carried out by scientists in lower-GDP nations. Our results highlight the need for a more systematic and directed evaluation of biodiversity studies, perhaps informing more targeted research towards those areas and taxa most depauperate in research. Only by doing so can we ensure that biodiversity research yields results that are relevant and applicable to all regions and that the information necessary for the conservation of threatened species is available to conservation practitioners.

## Introduction

**Fig 1:**
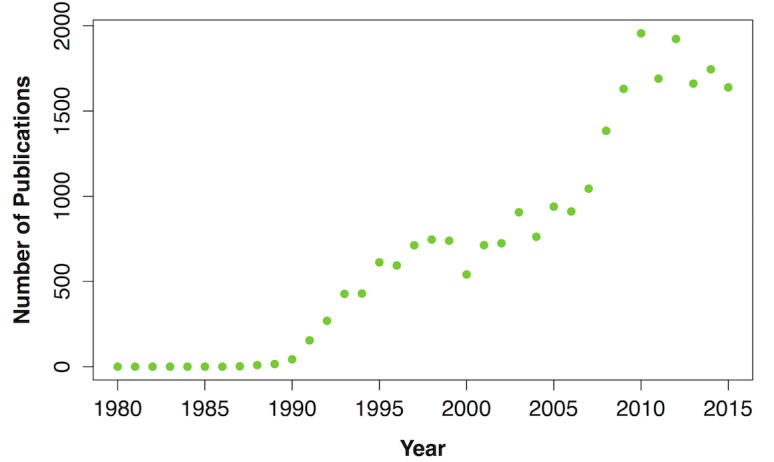
The number of papers whose title contains the word biodiversity over time from 1980–2015. A search for the word ‘biodiversity’ in Web of Science by year reveals the increase in biodiversity research over time (search date: 10^th^ February 2016).

Since 1988, when the word was first used in a publication [1], the idea of ‘biodiversity’ has become integrated into both popular and scientific culture. The word produces more than 50 million hits on Google [2] and almost 90,000 in the scientific search engine and database Web of Science at the time of writing [3]. Moreover, systematic quantification of the number of papers studying biodiversity shows a marked increase over the last two decades (Fig 1).

Biodiversity was formally defined at the 1992 United Nations Convention on Biological Diversity as ‘the variability among living organisms from all sources including, inter alia, terrestrial, marine and other aquatic ecosystems and the ecological complexes of which they are part; this includes diversity within species, between species and of ecosystems’[4]. The most commonly used meaning is diversity at the species level, although despite being an intuitive concept, in practice definitions of what constitutes a species, and estimates of Earth’s species richness, remain uncertain and variable. Estimates for global species richness typically fall in the range of 3 million to 100 million species [5] although a working figure between 5 and 15 million is often suggested [6].

Contrary to this uncertainly, it is well established that diversity is not evenly distributed amongst taxa. Arthropods, and especially insects, account for most known eukaryote species: of the 1.2–2 million described species, approximately 925,000 are insects [7,8]. However, it has become clear that public perceptions of biodiversity do not reflect this invertebrate-dominated reality. In the UK, children asked to draw their ‘ideal rainforest’ over-represented mammals, reptiles and birds, and under-represented insects and annelids [9]. Such taxonomic chauvinism is by no means restricted to children, nor is it restricted to non-academics: 31% of papers published in 2001 in three prominent conservation journals focussed on birds and mammals [10]. Although this focus on larger species is understandable, owing to their greater apparency and potentially greater importance for ecosystem processes and vulnerability to environmental change [11,12], it does mean that invertebrate conservation issues and extinctions may go unreported or unacknowledged. This could hamper an overarching understanding of the state of the natural environment. For example, only 70 modern insect extinctions have been documented, despite thousands being estimated to have occurred [13].

Several previous studies have examined these taxonomic biases in journal articles. A survey of papers on vertebrates from nine high-impact journals reported a bias towards mammals and birds [14]. Furthermore, mammal and bird studies had more ‘narrowly framed’ introductions and mentioned the study organisms sooner than in studies on fishes, reptiles or amphibians. In a review of fifteen years of research from two leading conservation research journals (*Biological Conservation* and *Conservation Biology*), an over-representation of vertebrates and under-representation of invertebrates was revealed [15]. Within vertebrates, birds and mammals were over-represented, while other taxa were under-represented. A similar study analysed the research in three prominent conservation journals [10], finding once again a weighting in favour of vertebrates, as well as towards pristine landscapes and single species, rather than communities. Another study focussed on the research output of four ecological journals (*Journal of Animal Ecology*, *Journal of Applied Ecology*, *Oecologia*, *Ecology*) for the years 2006 and 2007 [16], and again highlighted the tendency to ignore invertebrates, in particular insects, in high-impact journals. Also reported was a preference in British Research Council NERC funding towards vertebrate ecologists (38%) compared with entomologists (13%).

Thus, the topic of taxonomic seems well studied, although these four papers all used a similar approach, focussing on the research output of a few selected journals. In the present article, we take a different, more wide-ranging approach, sampling across the published literature for papers whose title contains the word biodiversity. We therefore do not discriminate by journal (hence nor by impact factor), aiming to obtain a more holistic and longer-term view of taxonomic biases in global biodiversity research. In addition, we chose to investigate geographical biases, to assess whether biodiversity research is skewed towards certain regions and whether taxonomic biases are stronger in certain parts of the world.

Specifically, we first investigate whether reported taxonomic biases (towards vertebrates, and towards birds and mammals especially) pervade papers on biodiversity and whether this weighting has changed over time. Secondly, we investigate whether any bias differs between temperate and tropical regions. Thirdly, we investigate how the titles of papers on biodiversity are phrased. In particular, whether papers studying biodiversity differ in how likely they are to specify the study taxon in the title compared between papers on invertebrate and vertebrate biodiversity. This may reflect and promote a common (if subconscious) perception of which taxa represent biodiversity. Fourthly, we investigate the global distribution of biodiversity research, compared to the actual distribution of biodiversity, to assess how well research effort reflects biodiversity. We also compare it to the distribution of IUCN Red-Listed species and GDP, to assess how research effort reflects conservation priorities and wealth. Finally, we investigate the authors’ countries of origin relative to the study location, to assess whether there is a mismatch between the distribution of research on biodiversity and biodiversity researchers by country.

## Materials & methods

### Sample selection

The scientific citation-indexing platform ‘Web of Science’ was used to sample research papers from the period 1995–2015, following a strict and repeatable search protocol. To be eligible for inclusion, papers’ title must have contained the word ‘biodiversity’, and also had to be a primary research article, in order to exclude review papers and other publication types such as books (which might have led to double-counting of studies). For each year, we then randomly selected 5% of all eligible articles using the random number generator *www.random.org* [17]. Five percent was an arbitrary figure that produced a sample size of 526 publications, which was quantifiable within the time frame of this project. This method may be cruder and return more irrelevant results than the careful examination of selected journals, but enabled us to easily generate a large sample size, and sample across a broad range of journals and disciplines over many years to obtain a comprehensive selection of biodiversity research. In this study we chose to focus on biases in animal biodiversity research, although we acknowledge that biases may also exist and be important across other taxonomic groups.

### Data collection

For each of the 526 papers in our sample, we recorded the taxon/taxa studied; the climate zone (temperate or tropical) in which the study took place; whether or not the taxonomic group was specified in the title; the country in which the study took place; the country of origin of the paper’s authors; and the number of times that paper had been cited as recorded in *Web of Science* at the time of searching. Vertebrate studies were classified into one or more of five major vertebrate groups (Mammals, Birds, Reptiles, Amphibians and Fishes). Correspondingly, five major invertebrate groups were chosen because of their high species richness and because they are relatively well studied (Insects, Arachnids, Nematodes, Annelids, and Molluscs). Studies on invertebrates that could not be classified into these five groups were recorded as ‘Other invertebrates’. When recording the climate zone, we considered any studies taking place between the Tropics of Cancer and Capricorn (23.5°N and S respectively) as ‘tropical’. Since only six polar studies existed in the sample, there were not enough to include these as a separate climate zone. We therefore considered all studies taking place at latitudes higher than the tropics to be ‘temperate’. By this classification, studies in polar regions are also classified as temperate. For each author, their country of origin was recorded as the country of their affiliated institution. If a paper had multiple authors from different countries, multiple countries were recorded for the authors’ country of origin.

### Data analysis

Statistical analyses were performed using R (version 3.0.2) [18]. To analyse the top 25% most-cited papers separately, the average number of citations per year was calculated (total citations to date divided by the time since publication). Chi-square tests were used to test for differences between temperate and tropical regions, and whether taxa were specified or not in the title. Wilcoxon rank-sum tests were used to test for differences between vertebrate and invertebrate residuals when comparing taxa for the proportion of studies versus proportion of described species as listed on the International Union for the Conservation of Nature (IUCN) database. Generalised linear models were used to test whether the number of biodiversity studies or authors in a country was related to Gross Domestic Product (GDP)–data from *World Bank*: *World Development Indicators 2014*. Maps were created using QGIS (version 2.12.1) to visualise differences in research effort across countries worldwide. In particular, we mapped the number of biodiversity publications per 1000km^2^ on vertebrates and invertebrates for each country, to visualise biases in research effort. We also mapped the number of authors relative to each country’s human population. By dividing the number of threatened species (data from IUCN [19]) by the number of biodiversity papers for each country, we also visualised countries that could be considered priorities for research (high numbers of threatened species relative to biodiversity research effort). Finally, analysis of covariance (ANCOVA) was used to test whether tropical and temperate regions differed in research effort for a given level of species or threatened species.

## Results

### Taxonomic biases

**Fig 2:**
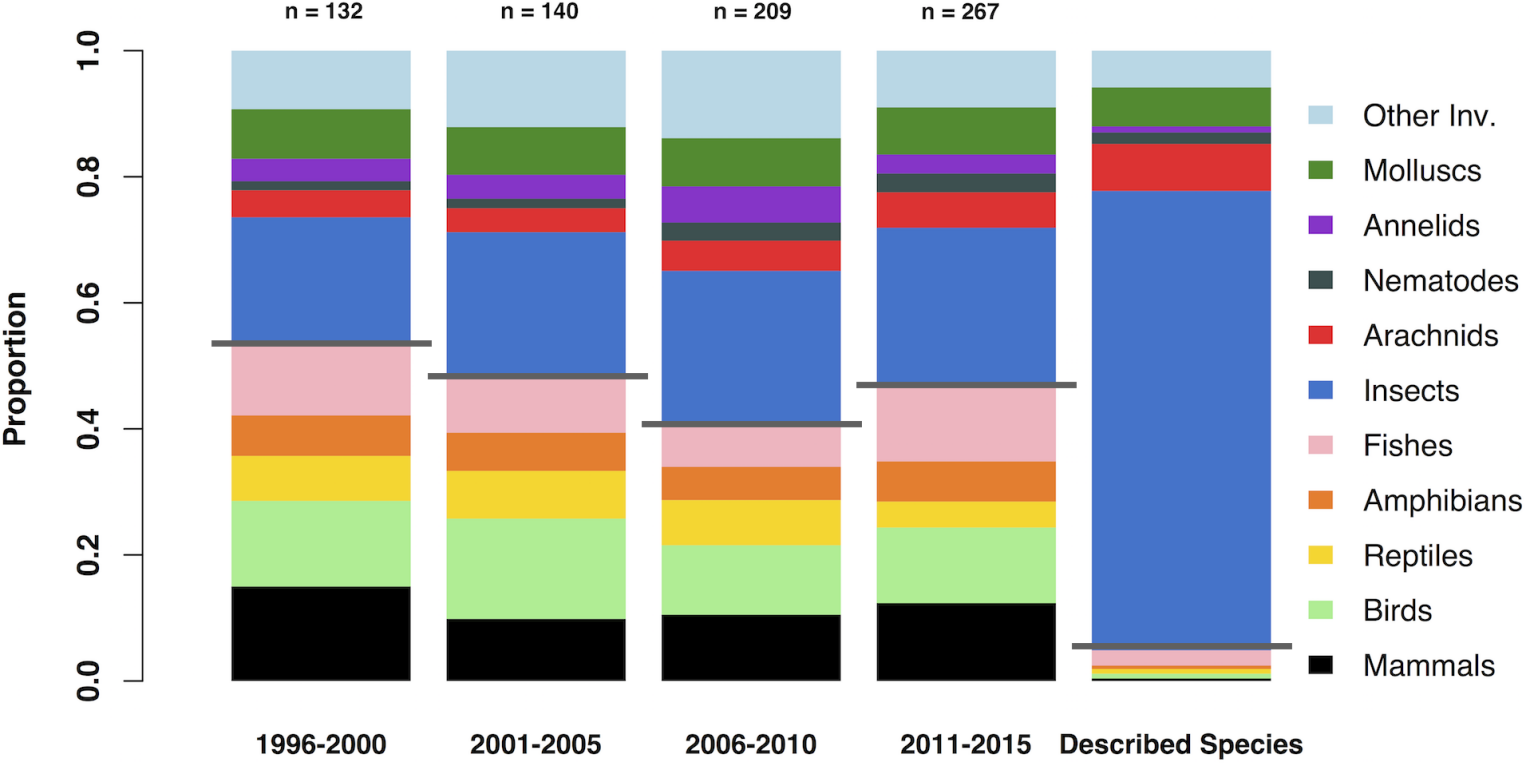
The proportions of different animal taxa studied in biodiversity research over the last 20 years. The proportion of different taxonomic groups in the sample of papers with ‘biodiversity’ in the title is shown for 4 five-year periods since 1996. For comparison, the right-hand column illustrates the ‘true’ proportions of described species that each group makes up (data from IUCN [20]) Vertebrate and invertebrate taxa are separated by a grey line.

**Fig 3:**
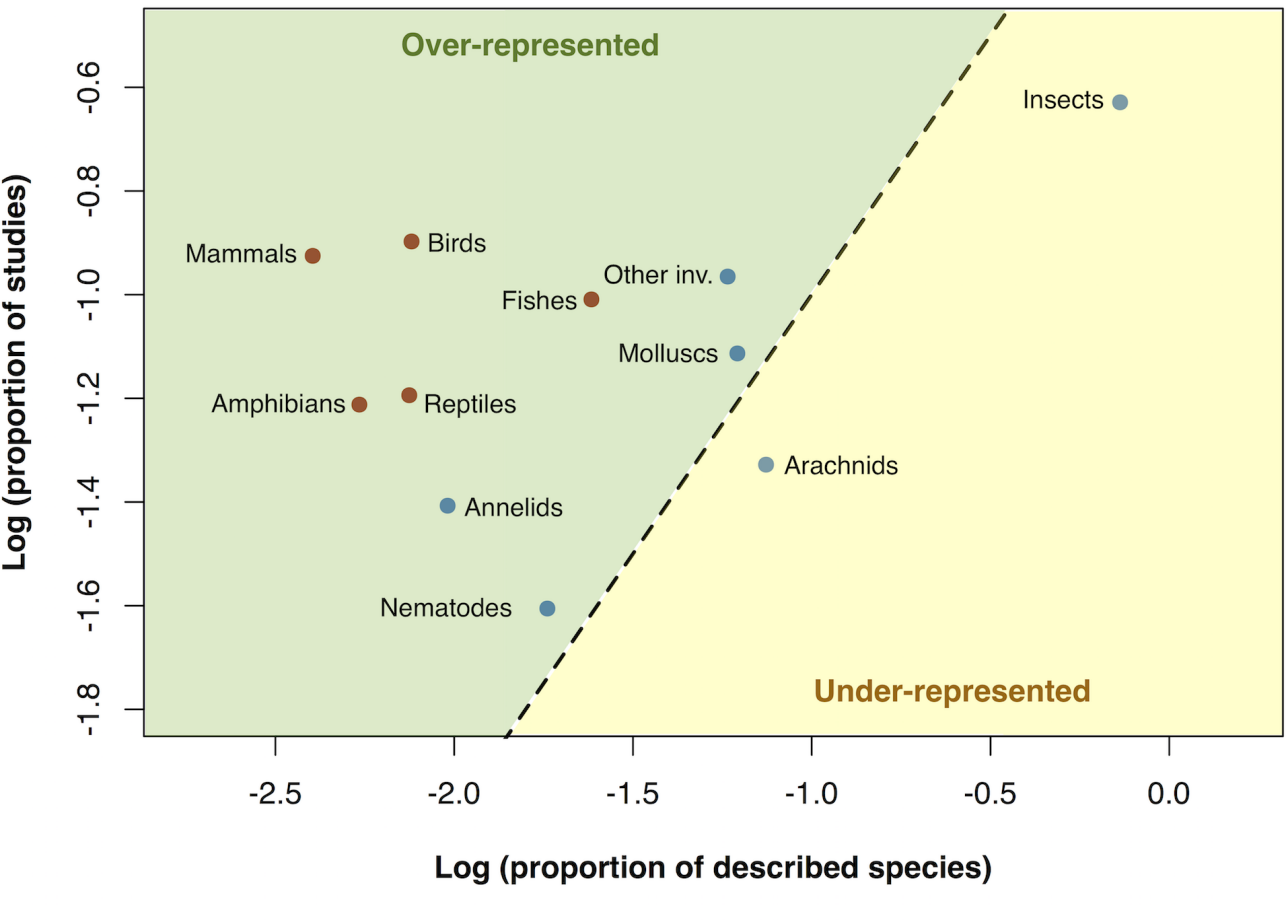
The over- and under-representation of different animal groups in biodiversity research relative to the number of described species. The proportion of studies on each taxonomic group is plotted against the ‘actual’ proportion of described species [20] found in that taxon. Values were log transformed for clarity. The 1:1 line is shown (dotted); over-represented groups are found above the line while under-represented groups are below it. Vertebrate groups are shown in red and invertebrate groups are shown in blue.

Approximately half of the papers sampled studied vertebrates, and half studied invertebrates (Fig 2). However, this is far from the true proportions of described species, where over 95% of species are invertebrates (see right-hand column of Fig 2). Furthermore, this focus on vertebrates has been roughly consistent over the last 20 years. Given their true species richness, vertebrates were significantly over-represented compared to invertebrates in the published literature (Wilcoxon rank-sum test, W = 24, N = 10, P<0.05) (Fig 3). Invertebrate taxa were either slightly over-represented (annelids, molluscs, nematodes and ‘other invertebrates’) or under-represented (insects and arachnids). In addition, the taxonomic bias was greater in highly cited papers. Of the top 25% most cited papers in the sample, only 47% included invertebrates, compared with 57% of the entire sample.

### Comparing tropical and temperate regions

**Fig 4:**
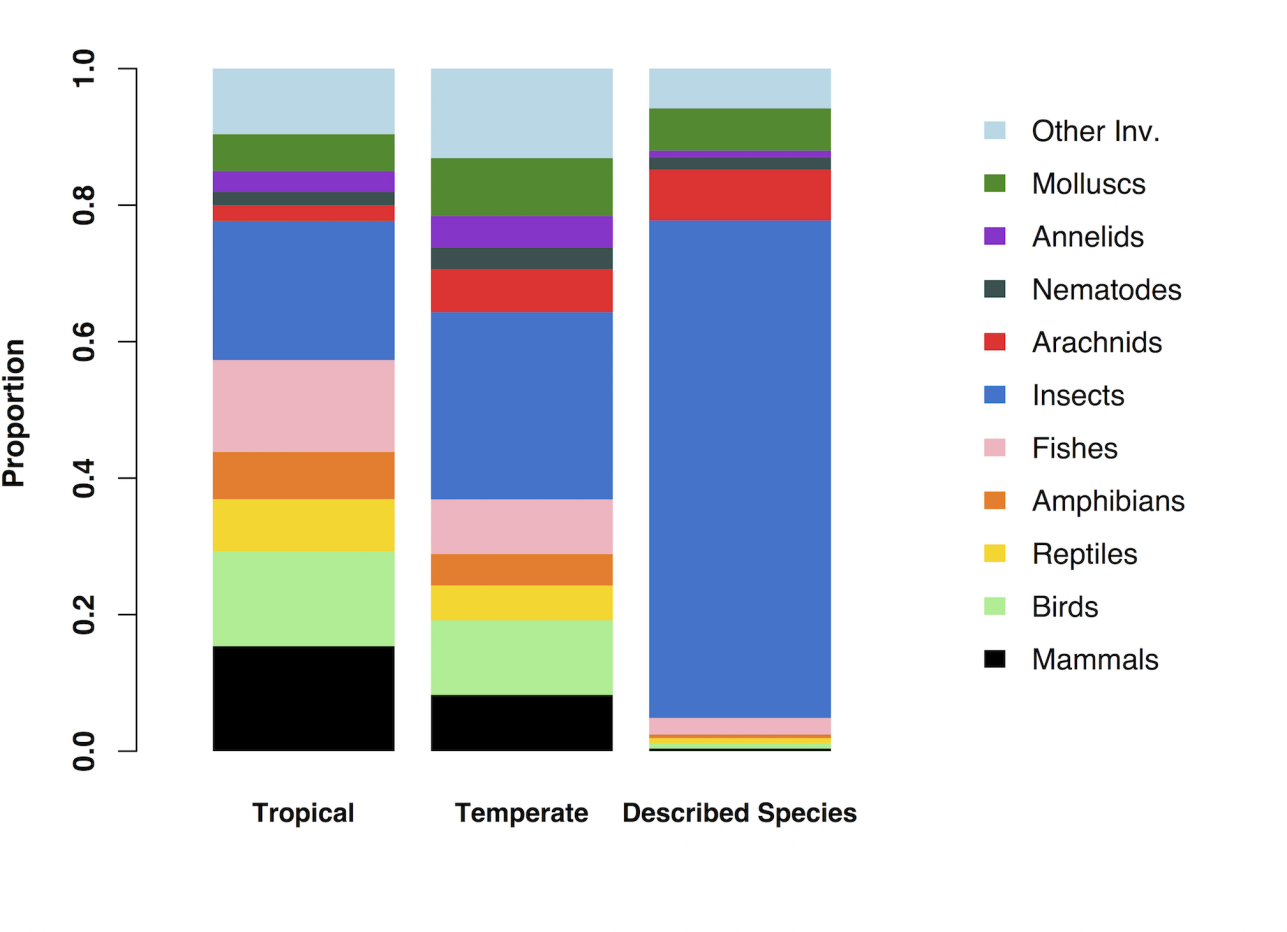
Representation of different animal groups in temperate and tropical biodiversity studies. The bias towards vertebrates is greater in tropical regions than temperate regions. The proportions of described species in different groups are shown in the right-hand column for comparison.

In terms of the proportion of studies, the bias towards vertebrates was greater in tropical regions than temperate regions (Chi-square test, X^2^ = 30.65, N = 672, P<0.001) (Fig 4). In tropical countries, 43% of studies included invertebrates, compared to 63% in temperate countries. General patterns of taxonomic over- or under-representation were similar in tropical and temperate regions, although arachnids were particularly under-represented in the tropics, and molluscs were under-represented in the tropics despite being over-represented in temperate studies.

### Differences in title phrasing

**Fig 5:**
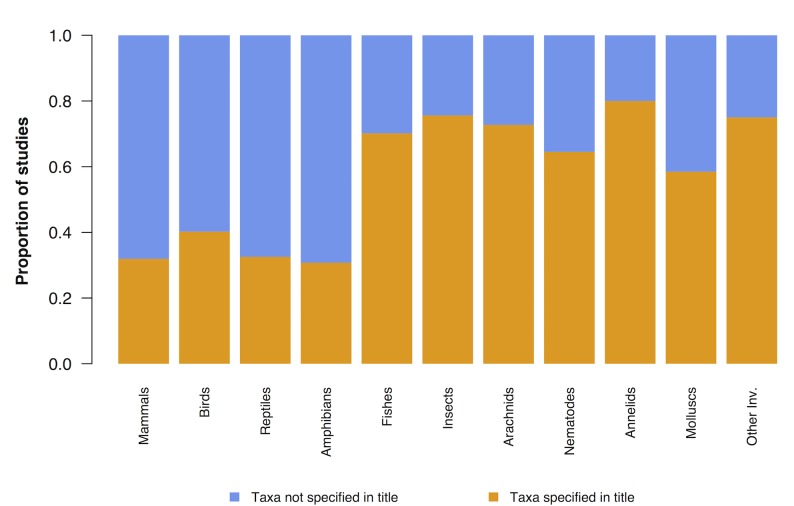
The phrasing of papers’ titles differs between taxonomic groups. The majority of studies on vertebrates (with the exception of studies on fishes) do not mention the study taxon in the title. Conversely, for papers on invertebrates, the taxa being studied were specified more often than not.

The proportion of papers for which a taxonomic group was specified in the title differed between vertebrates and invertebrates (Chi-square test, X^2^ = 103.45, N = 714, P<0.0001) (Fig 5). Specifically, most papers that studied vertebrates did not specify the study taxon/taxa in the title, and instead referred to ‘biodiversity’ more generally. In contrast, the titles of studies on invertebrates usually specified which taxa were being studied. An exception to this pattern was studies on fishes, where the majority of studies specified the taxon in the title.

### Geographic biases

**Fig 6:**
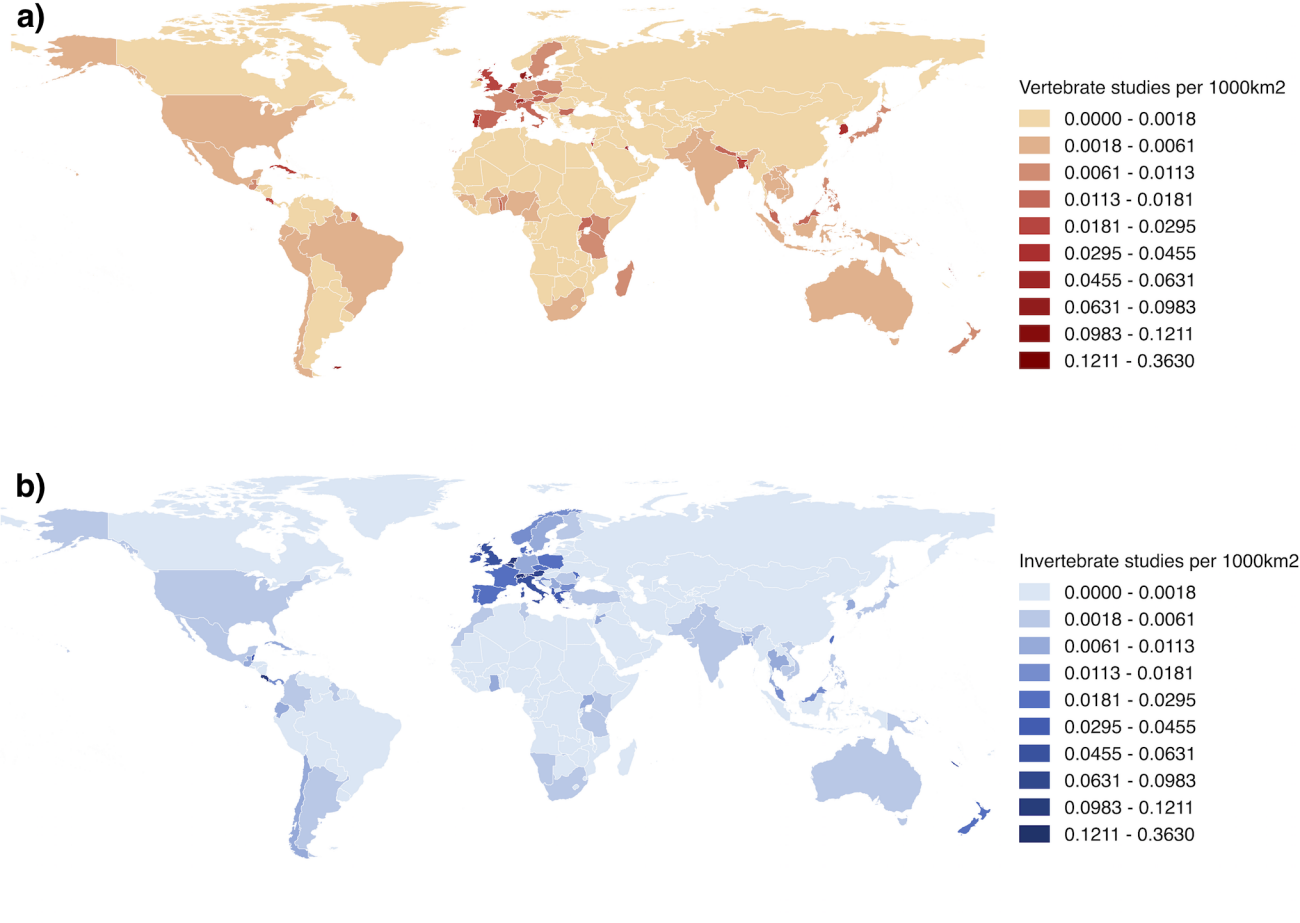
The global distribution of biodiversity research by country. The number of papers with ‘biodiversity’ in the title per 1000km^2^ is shown, for a) papers that study vertebrates and b) papers that study invertebrates. Darker colours represent a higher density of studies.

**Fig 7:**
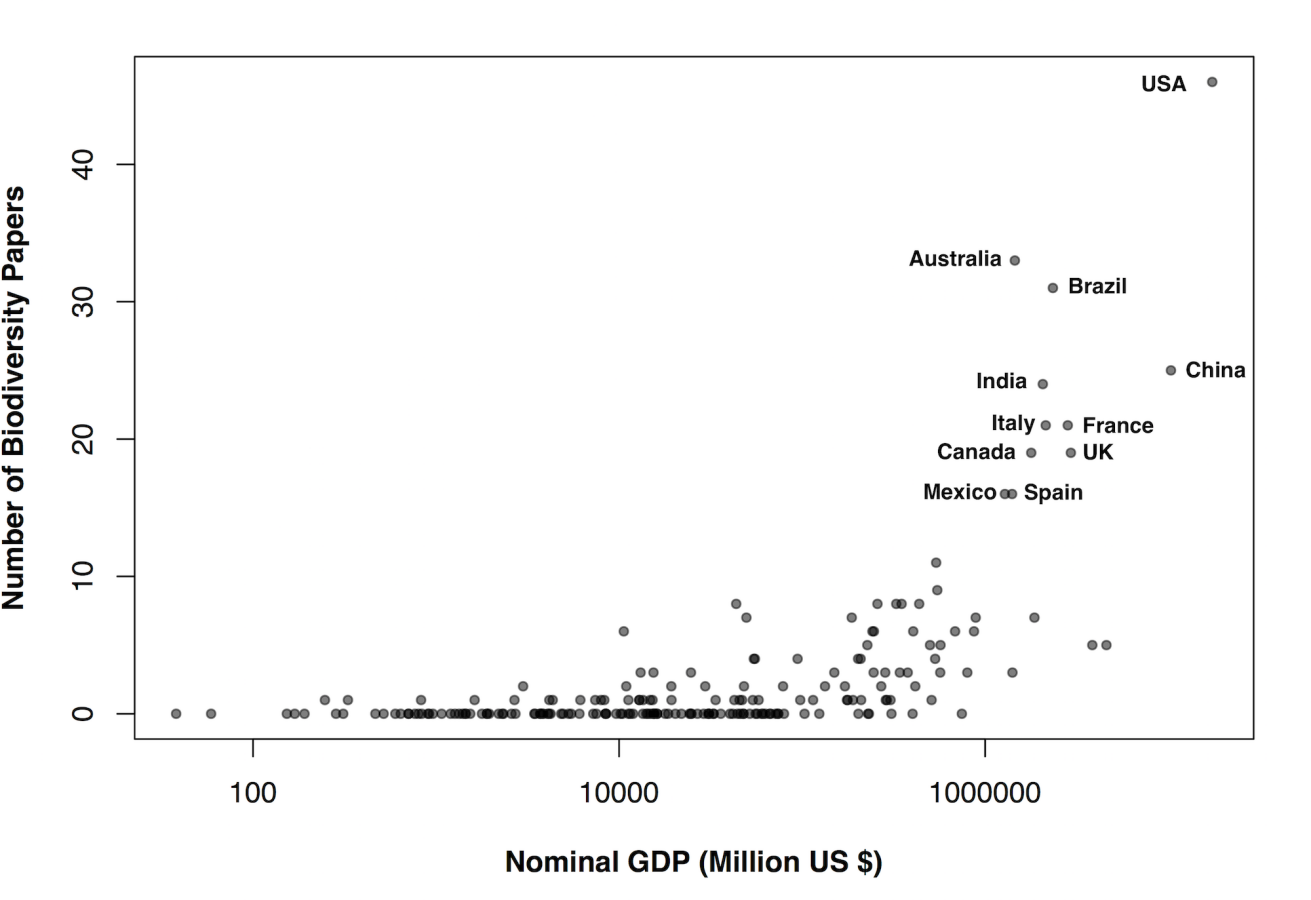
The number of biodiversity papers in a country related to its GDP. Nominal GDP in US$ is plotted against the number of biodiversity studies sampled from each country, revealing a positive relationship. The top ten countries for number of papers are labelled. Many countries with low GDP had no biodiversity papers identified from this sample.

Biodiversity research was more commonly carried out in developed countries with larger economies, for both vertebrate and invertebrate studies (Fig 6). The United States of America had the highest number of studies of any country in the sample, but the density of biodiversity research appears to be generally highest in Western Europe. Most tropical areas had fewer studies and very little research was based in African countries. The number of biodiversity studies was positively related to countries’ nominal GDP (Poisson regression, z = 28.62, N = 232, P<0.0001) (Fig 7).

**Fig 8:**
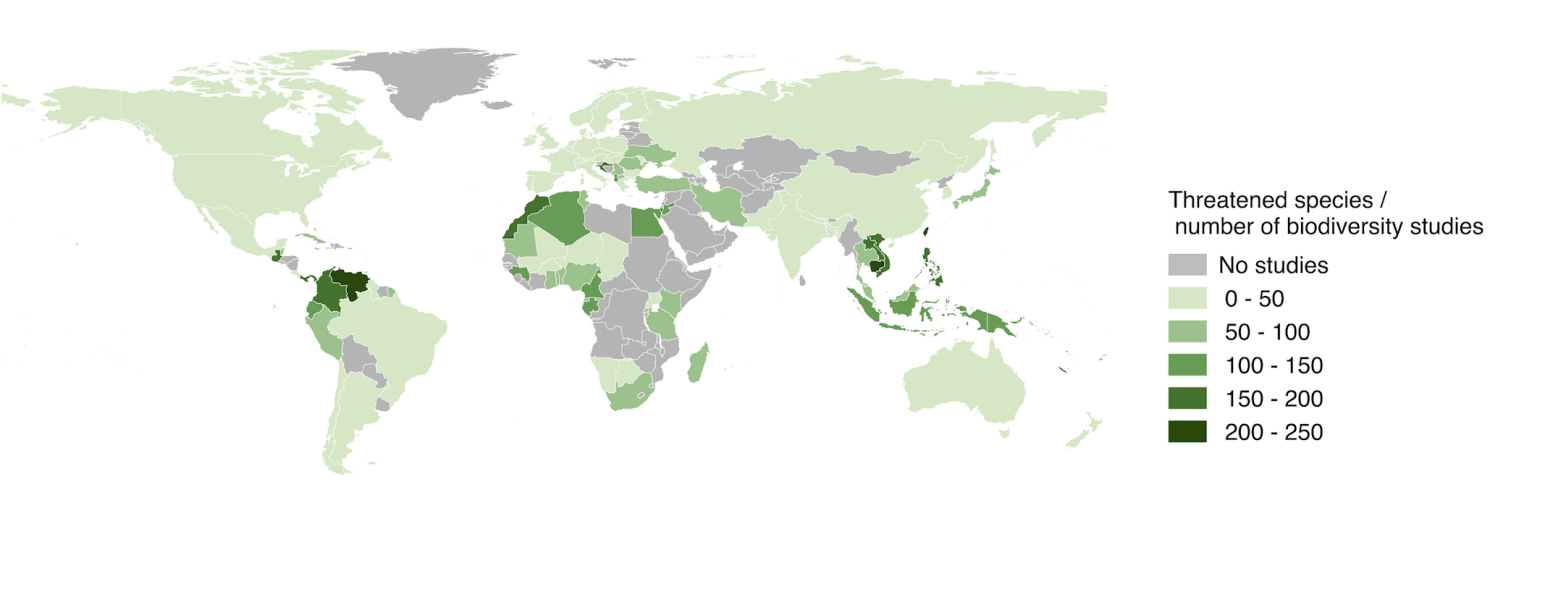
The number of Red-Listed animal species in each country relative to the number of biodiversity studies. Dividing the number of animal species threatened with extinction [19] by the number of biodiversity studies reveals regions that are understudied given their number of threatened species. Countries in northern South America, Africa and SE Asia stand out as being relatively understudied; much of central Africa lacked studies altogether in this sample. Darker colours represent a higher number of listed threatened species per study.

**Fig 9:**
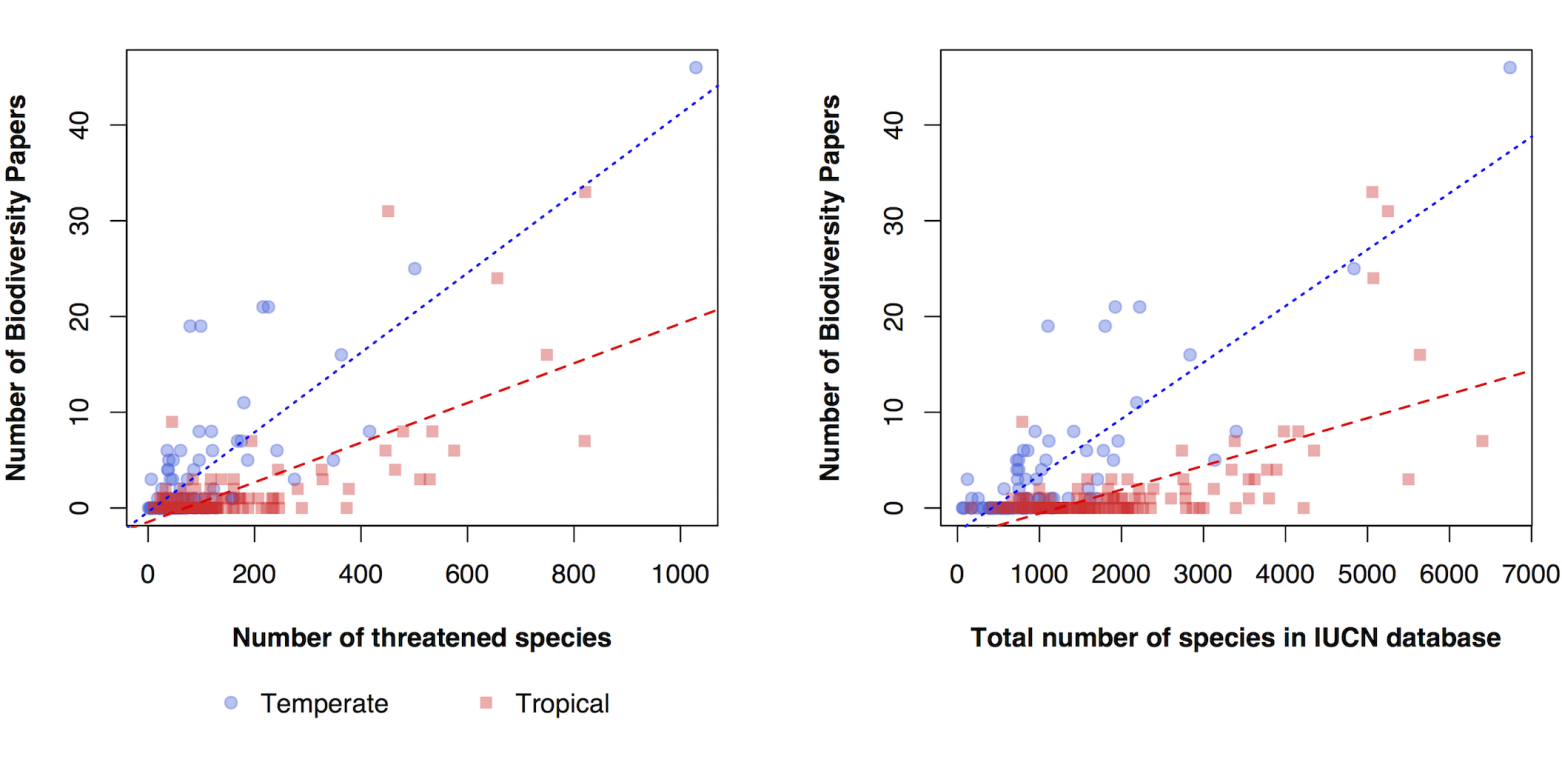
**Scatterplots comparing the number of biodiversity papers against the number of threatened animal species (a) and species richness (b) listed in IUCN databases [19, 20] per country.** Temperate countries tend to have more biodiversity research than tropical countries for a given number of threatened species or a given species richness.

Certain counties had a higher number of threatened species relative to the biodiversity research effort (given by dividing the number of IUCN listed threatened species [19] by the number of research publications on biodiversity (Fig 8). In particular, northern South America, Africa and SE Asia had a low relative number of publications. Note that large areas of Africa lacked any studies at all in our sample. We recorded a generally a positive relationship between the number of publications and the number of threatened and number of species recorded in the IUCN database [19, 20] per country. However, for a given level of species or threatened species, tropical regions were understudied compared to temperate regions; interactions were significant between climate region and number of threatened species (F_3,227_ = 36.06, p<0.0001) (Fig 9A) and between climate region and number of species (F_3_,_227_ = 48.28, p<0.0001) (Fig 9B).

**Fig 10:**
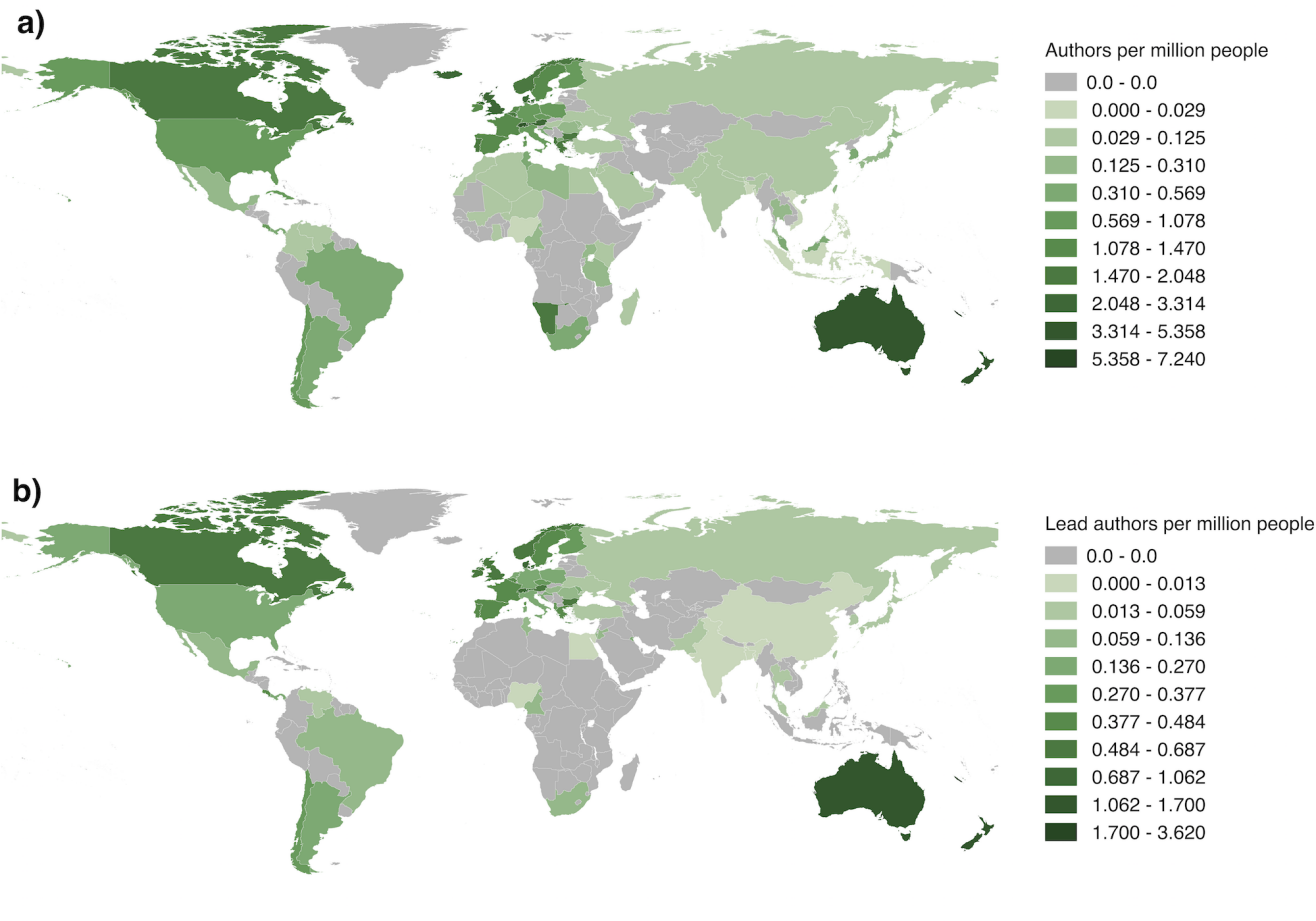
Number of authors and lead authors per million people for each country. The number of authors (a) and lead authors (b) from each country relative to the country’s population. Many countries in Africa, central Asia and South America lacked authors on the papers in the sample.

As with the distribution of biodiversity research, the distribution of authors was heavily biased towards developed countries, particularly Western Europe (Fig 10). Many countries in Africa, central Asia and South America lacked any authors on the papers in the sample; this is particularly true when looking at lead authors only (Fig 10B). The number of authors from a country was strongly related to wealth of that country as approximated by nominal GDP (Poisson regression, z = 69.91, N = 232, P < 0.0001). Furthermore, the GDP of authors’ countries of origin (median 2,066,902 million US$) was significantly higher than the GDP of study locations (median 1,453,770 million US$) (Wilcoxon rank-sum test, N = 513, W = 89086, P < 0.0001).

## Discussion

### The taxonomic bias

These results clearly demonstrate more charismatic animal groups are over-represented in biodiversity research and have been since biodiversity first emerged as a research field. Mammals, which make up around 0.4% of known animal species [20], were studied in approximately 12% of papers with biodiversity in the title. The equivalent numbers for birds are 0.7% and 13%. In contrast, insects make up at least 70% of animal species [20] yet were studied in less than a quarter (23%) of papers. This result corroborates earlier findings [10,14–16], and extends the phenomenon to all biodiversity research rather than just that of selected journals. Due to the high proportion of species remaining to be described, particularly among the invertebrates, this figure is likely to be conservative. These results have implications for awareness of the natural world in the scientific community, particularly as this taxonomic bias was greater in the top quartile of most-cited papers, suggesting that the research with the highest impact and largest influence is even less representative of the real world in this regard.

The taxonomic bias was greater in tropical regions, where vertebrates were studied in more than half of papers, despite vertebrates comprising less than 5% of animal species. As tropical countries contain a higher total species number and are therefore likely to have a much higher proportion of undescribed species [5], particularly smaller taxa, this under-representation is likely to be even more marked in reality. Ensuring adequate research coverage across taxa in tropical regions has important conservation implications. Most species are found in the tropics [21] and tropical regions encompass many of the world’s conservation priority hotspots [22], but are currently experiencing habitat loss faster than any other region [23].

Not all invertebrate taxa were underrepresented however; in fact, four out of the six invertebrate groups were somewhat over-represented in scientific research. The overall lack of invertebrate studies is, more precisely, a dearth of global insect and arachnid research and tropical mollusc research. The fact that insects and arachnids were the least well represented groups in this study does not mean they are the least represented of *all* taxa, since there will be other poorly studied invertebrate groups included within the *other invertebrates* category, or within these groups at a finer taxonomic scale. However, since arachnids and insects are so speciose, the deficiency of research in these groups is perhaps most significant to understanding global biodiversity. Another key finding relating to taxonomic bias is that studies on vertebrates typically did not specify the taxon in the title, referring to ‘biodiversity’ more generally. This was not the case for invertebrate research, for which the study taxa were usually specified. This could reflect a general perception that vertebrates alone are sufficient to represent biodiversity.

This unequal coverage of research across taxa may have a complex combination of causes. Researchers themselves may find studying charismatic vertebrates more appealing. Alternatively, it could represent the increased challenges of working with more diverse taxa, particularly in terms species identification. This is despite studies showing that certain insect groups are informative indicators of biodiversity and cost effective taxa to sample [24,25]. General perceptions of biodiversity may also be influenced by journal editors publishing a disproportionate number of articles on vertebrates (consciously or subconsciously), because such articles may be more likely to gain traction within a scientific community that is already vertebrate-biased (especially if journals are under pressure to maintain a high impact factor driven by citations). Vertebrate-biased research may also appeal to the media who are catering for a vertebrate-preferring public audience [9]. The taxonomic bias could also be the product of funding bodies, which may preferentially award research grants for vertebrate studies if these are perceived to be more important, interesting or relevant to conservation and policy priorities. A few or all of these hypotheses may play a role in producing the biases reported in this study.

Taxonomic bias is not necessarily bad. A bias towards charismatic vertebrate taxa may be advantageous where such taxa have a disproportionately large role in ecosystem functioning (keystone species), in generating funds and support for conservation (flagship species), or when their protection also ensures the protection of much of their ecosystem (umbrella species) [26,27]. In addition, certain taxa may be used as surrogates for other harder-to-study groups [28,29], which may have a similar geographic distribution or show a similar response to disturbance. However, notwithstanding doubt over the prevalence of keystone species and the reliability of taxonomic surrogates [30,31], it is unlikely that the taxonomic bias we have observed has arisen as a result of deliberate decisions to select these taxa as indicators of other lesser-known animal groups.

In using the proportion of described species as a reference for many of our analyses, we implicitly make the assumption that all species are equal. However, clearly this is not the case in terms of ecosystem function or conservation priority. It would be interesting to investigate whether the proportion of research done on different taxonomic groups better reflects the distribution of ecological importance or conservation value among taxa (rather than the proportion of described species), but it remains a challenge to identify meaningful measures for these that are comparable across taxa and globally applicable [32].

### The geographic bias

The distribution of biodiversity research and its authors’ countries of origin resemble the distribution of GDP, rather than that of actual biodiversity or numbers of threatened species. The distribution of research is skewed towards developed countries and particularly Western Europe. Furthermore, even when studies are carried out in lower GDP-countries, the authors tend to be based at institutions in wealthier nations. Tropical countries tend to have fewer biodiversity studies despite being where more biodiversity is found and where biodiversity is most threatened. Tropical regions were also where the taxonomic bias was greatest. Taken together, these findings have important implications for biodiversity conservation: the same areas that are most threatened and most diverse are the least studied [23] and where scientists research is most skewed towards less-speciose groups. Therefore, we are likely to continue to undervalue these under-studied groups, especially in parts of the world where they are most threatened, and perhaps allocate less funding to their protection. Moreover, given that conservation efforts will be more likely to succeed when we better understand the target organisms, there is a real possibility that we may be ill equipped to protect the majority of animal biodiversity. Research gaps may mean we are less likely to identify threatened invertebrates and notice their disappearance, and we may be less likely to identify underlying threats and their drivers. Furthermore, without a good understanding of invertebrate biodiversity loss, we may suffer a reduced ability to predict subsequent anthropogenic impacts on ecosystems worldwide. Given that funding and time are limited, biodiversity research should be focussed on certain taxa for scientifically justified reasons, rather than because of an underlying subjectivity in what we consider to be important. Crucially, conservationists need to be more aware of these unequal weightings to prevent biodiverse taxa being overlooked or understudied.

### Redressing biases

Significant challenges remain in addressing the biases we found. One is to popularize these lesser-known taxa to allow recognition of their importance. This could be achieved through more targeted funding for these invertebrate groups (and under-represented countries). Another challenge is to ease the practical issues of identification and research on these taxa [33]. Opportunities may be found in novel techniques such as metagenomic sequencing [34], or the development of apps that aid easy identification worldwide [35]. The use of modern media may ease access to specimens digitally, and help to put researchers and taxonomic experts in touch. It will require a concerted effort to redress these research biases and to ensure the least studied taxa and countries do not remain so, thus ensuring that we maximise the contribution of biodiversity research to our understanding of nature, and minimise the further erosion of biodiversity in our increasingly imperiled world.

## Supporting information

S1 Dataset Data used in this study. See ‘metadata’ sheet for more information.
(XLSX)
